# Selenoprotein P Controls Oxidative Stress in Cornea

**DOI:** 10.1371/journal.pone.0009911

**Published:** 2010-03-29

**Authors:** Akihiro Higuchi, Kazuhiko Takahashi, Masaki Hirashima, Tetsuya Kawakita, Kazuo Tsubota

**Affiliations:** 1 Center for Integrated Medical Research, School of Medicine, Keio University, Tokyo, Japan; 2 Department of Nutritional Biochemistry, School of Pharmacy, Hokkaido Pharmaceutical University, Hokkaido, Japan; 3 Research Department 1, The Chemo-Sero-Therapeutic Research Institute, Kumamoto, Japan; 4 Department of Ophthalmology, School of Medicine, Keio University, Tokyo, Japan; Johns Hopkins University, United States of America

## Abstract

The ocular surface is always attacked by oxidative stress, and cornea epithelial cells are supposed to have their own recovery system against oxidative stress. Therefore we hypothesized that tears supply key molecules for preventing oxidative stress in cornea. The potential target key molecule we focused is selenoprotein P (SeP). SeP is a carrier of selenium, which is an essential trace element for many animals, for oxidative stress metabolism in the organism, and was extremely expressed in lacrimal gland. An experiment was performed with SeP eye drops in a rat dry eye model, prepared by removing the lacrimal glands. The anticipated improvement in corneal dry eye index and the suppression of oxidative stress markers were observed in SeP eye drop group. Furthermore, the concentration of SeP was significantly higher in dry eye patients compared with normal volunteers. Collectively, we concluded that tear SeP is a key molecule to protect the ocular surface cells against environmental oxidative stress.

## Introduction

Ocular surface cells were most challenging cells for oxidative stress, direct contact with the airflow, and light exposure including ultraviolet irradiation. However eyes need to open and expose to such environments for acquiring vision. To overcome such environments, ocular surface cells (corneal and conjunctival epithelial cells) are covered with tear film. If tear film lost their balance of their components, tear film decrease their stability and resulted in exposed directly in oxidative stress with dryness. Although the severe case of dry eye syndrome was vision-threatening diseases, and the number of patients is still increasing, their molecular mechanism was poorly understood.

Tear film contains many kinds of working components [1] which protect ocular surface cells against physical factors including temperature [2], humidity [3], ultraviolet irradiation [4], and airflow [5]. However no defined molecule was identified related to the mechanism of dry eye syndrome. Among dry eye syndrome, Sjogren's syndrome is the well researched disease for dry eye, and their ocular surface was dry due to decreased tear secretion from the lacrimal glands. Therefore their dry eye results not only from desiccation of the ocular surface, but also from the lack of tear components essential for maintaining the ocular surface [6]–[7]. This could be partially explained by that autologous serum for the treatment of severe dry eye resulted in dramatic improvement in the ocular surface [8], but artificial tears could not. The remarkable results using autologous serum led us to investigate whether serum contains defined factors for the treatment of dry eye.

Recently, dry eye was recognized as oxidative stress-induced diseases. Selenium is an essential trace element for the human body. Low selenium states have been associated with numerous diseases, such as viral infection, reproductive dysfunction, cardiovascular disease [9], and cancer [10]. SeP deficient mice showed low level of selenium in brain, and short life span, rescued by a high selenium diet. [11] For *in vitro*, selenium was necessary in many types of cells, and promoted proliferation [12], which was mainly supplied by fetal bovine serum (FBS) in culture medium. Collectively, SeP, isolated through human serum fractionation, was identified as key molecule for dry eye syndrome, and also another candidate for treatment of dry eye.

## Materials and Methods

### Evaluation of oxidative stress in rat dry eye model

All animal experiments as follows were approved by the Laboratory Animal Care and Use Committee of Keio University School of Medicine. The dry eye rat model was prepared by unilateral removal of the lacrimal gland [13]. Male 6-week-old Sprague-Dawley rats (n = 10 in each experiment) were purchased from CLEA Japan, Inc. (Tokyo, Japan). The lacrimal gland was surgically removed from the anesthetized rats. To estimate the degree of dry eye, the ocular surfaces of rats were photographed after being stained by fluorescein for scoring as follows. The photographs of the corneas were divided into 9 areas. Each area was scored from 0 to 3 points depending on degree of staining, and the scores of all areas were totaled. This total was called the ‘fluorescein score’. Tear volume was measured with cotton thread as typical for clinical practice. The fluorescein score and tear volume for each rat were measured before treatment and after 1, 2, 3, and 4 weeks of treatment. After 4 weeks of treatment, the corneas were collected to measure 8-OHdG (8-hydroxy-2′-deoxyguanosine, a marker of oxidative DNA damage [14]) and HEL (*N^6^*-Hexanonyl lysine, an initial marker for oxidative damage [15]). 8-OHdG and HEL were measured by High Sensitivity 8-OHdG Check (Japan Institute for the Control of Aging, Fukuroi, Shizuoka, Japan) and HEL ELISA Kit (Japan Institute for the Control of Aging), respectively.

### Effects of SeP eye drops treatment in rat dry eye model

The effect of SeP on treatment for dry eye was estimated by applying SeP eye drops to the dry eye rat model. One eye in each dry eye rat was treated with 5 or 50 µg/mL SeP or PBS, and the other side was treated with PBS (n = 10 in each group). Five µl of each eye drops was administrated 6 times per day for 3 weeks. After all eye drop treatments, the corneal fluorescein scores were estimated and the corneas were collected to measure the 8-OHdG concentration. To avoid observer bias, the components of each eye drops was kept blinded until after statistical analysis.

### The measurement of oxidative stress in selenium-deprived CEPI cells

To examine the influence of selenium deficiency on cellular response against oxidative stress, we prepared selenium-deprived cells using a selenium-deficient medium. Human corneal epithelial cell line, CEPI-17-CL4 (CEPI) cells, was kindly provided by Dr. Kuwahara (Alcon Laboratories, Fort Worth, TX). These immortalized cells were infected with a recombinant SV40-retrovirus vector containing the Bg1I-HpaI fragment of SV40 T-antigen; they express an extensive array of cytokines, growth factors, and metabolic enzymes that resemble the original tissue [16]. CEPI cells were cultured in serum-free medium (EpiLife supplemented with HCGS; Cascade Biologics Inc., Portland, OR) with (Se+) or without selenium (Se−). Selenium deficiency was monitored every week by measurement of glutathione peroxidase activity in the cell lysate. GPx is a well-characterized selenoprotein, in which the selenium atom is essential to catalyze the enzyme reaction. After GPx activity diminished, 0, 0.5, 5, or 50 µg/mL SeP was added to the medium and the cells were incubated 0, 1, 2, or 3 days to allow uptake of SeP into the CEPI cells. To estimate the influence of SeP uptake in CEPI cells during oxidative stress, GPx activity and the 8-OHdG and HEL content were measured. GPx activity was measured using a GPx assay kit (Cayman Chemical Company, Ann Arbor, MI, USA). The concentrations of 8-OHdG and HEL in the cells were measured using a High Sensitivity 8-OHdG Check and HEL ELISA Kit, respectively.

### Effect of SeP against oxidative damage in selenium-deprived CEPI cells

CEPI cells were grown under normal conditions to 80–90% confluence in 96-well plates. To assay the production of hydroperoxide in CEPI cells, 0.25 mM diphenyl-1-pyrenylphosphine (DPPP) was added to the medium and the cells were incubated for 24 hours. DPPP is a fluorescent reagent for detection of hydroperoxide (Dojindo Laboratories, Mashikimachi, Kumamoto, Japan). Cumene hydroperoxide (0.2 mM) was added to the medium to cause oxidative damage to CEPI cells. SeP was co-incubated with cumene hydroperoxide to protect against production of hydroperoxide. Lipid peroxidation in the cells was measured by Plate Reader (ARVO SX; PerkinElmer Japan Co., Ltd., Yokohama, Kanagawa, Japan).

### SeP in human tears from dry eye patients

This study followed the guidelines of Institutional Review Board and of the tenets of the Declaration of Helsinki. All study participants signed informed consent documents prior to participation. Two µl of human tears were obtained from healthy volunteers (n = 9) and dry eye patients (n = 49) using capillary collection. Tears were diluted approximately 240-fold by PBS with 0.05% Tween 20. The concentration of SeP was measured by sandwich EIA. Dry eye was diagnosed according to published criteria [17].

### SeP expression in rat lacrimal gland

Corneas, hearts, kidneys, lacrimal glands, livers, muscles, pancreases, and salivary glands were removed from rats (n = 6). Total RNA was extracted by GeneElute (Sigma-Aldrich Co.; Chicago, IL, USA), and SeP expression was measured by real time RT-PCR (ABI PRISM 7700 Sequence Detection Systems; Applied Biosystems, Inc.; Foster City, CA, USA). All data were analyzed with ΔΔCt methods according to the manufacturer's protocol.

## Results

### Removal of lacrimal gland affected tear volume and corneal damage, and increased oxidative stress

**Figure 1:**
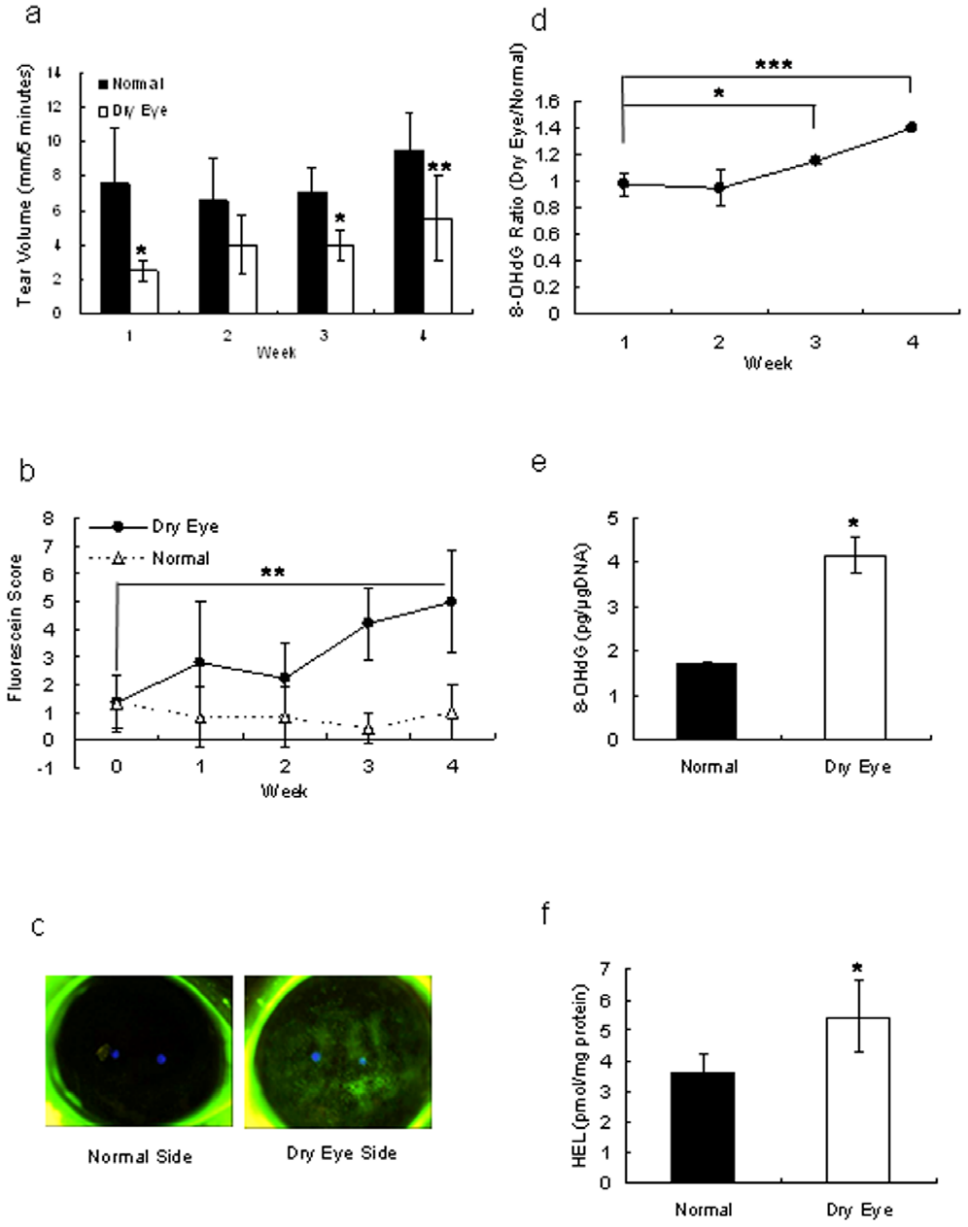
Preparation of dry eye rat model. a: Measurement of tear volume secreted from normal eye or lacrimal gland-removed eye of rats. The vertical axis shows tear volume measured by cotton thread. Results are expressed as mean ± S.D. Dunnett's test was used to determine the significance of differences. * and **indicates a significant difference from the result in normal side, *P*<0.05 and <0.01, respectively. b: Corneal fluorescein staining in normal rats and rats with dry eye. The vertical axis shows score of the fluorescein-stained area. Dunnett's test was used to determine the significance of differences. ** indicates a significant difference from the result at 0 week, *P*<0.01 (n = 10). c: Photo of cornea stained by fluorescein after 4 weeks treatment. d: Increase of marker during oxidative stress. Ratio of 8-OHdG content in the cornea between the dry eye and normal eye. Results are expressed as mean ± S.D. Dunnett's test was used to determine the significance of differences. * and *** indicates a significant difference from the result in 1-week removal, *P*<0.05 and <0.005, respectively. e, f: Oxidative stress markers in cornea of normal rats and rats with dry eye. Contents of 8-OHdG (e) and HEL (f) in a dry eye and normal corneas. t-test was used to determine the significance of differences. * indicates a significant difference from the result in normal side, *P*<0.05. Results are expressed as the mean ± S.D. (n = 10).

Unilateral removal of lacrimal glands led to decreased tear secretion (Fig. 1a), and resulted in corneal erosion on the dry eye side. Fluorescein scores for the dry eye corneas gradually increased, while the cornea on the untreated side did not change (Fig. 1b). Four weeks after removal of the lacrimal glands, the fluorescein-stained areas on the dry eye side were larger than on the normal side (Fig. 1c). The amount of 8-OHdG measured on the dry eye side started to increase 3 weeks after removal of the lacrimal gland (Fig. 1d). Four weeks after removal of the lacrimal gland, the amounts of 8-OHdG (Fig. 1e) and HEL (Fig. 1f) increased in the corneas of the dry eye side. Removal of the lacrimal gland led to an increased fluorescein-stained area accompanied by an increase in oxidative stress.

### SeP partially recovered oxidative damage of cornea

**Figure 2:**
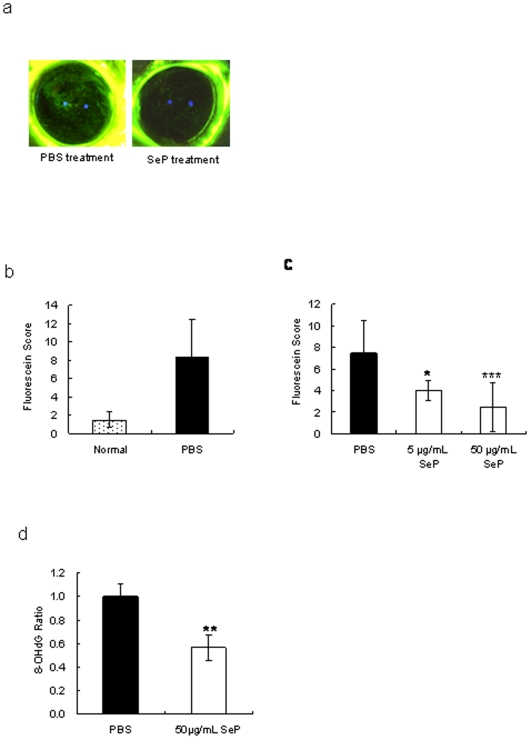
Effects of SeP eye drops on the cornea of a dry eye rat model. a: Photo of cornea stained by fluorescein after 3 weeks of SeP eye drop administration. b: Fluorescein score of cornea in normal rats (Normal), in dry eye rats treated with PBS (PBS, closed bar). Results are expressed as mean ± S.D. Dunnett's test was used to determine the significance of differences. * and *** indicates a significant difference from the result in PBS treatment, *P*<0.05 and <0.005, respectively. c: Fluorescein score of cornea in dry eye rat treated with PBS or SeP. d: Ratio of 8-OHdG content in cornea of dry eye between SeP and PBS treatment. Results are expressed as mean ± S.D. T-test was used to determine the significance of differences. *, **, and *** indicates a significant difference from the result in PBS treatment, *P*<0.05, <0.01, and <0.005, respectively (n = 10).

Identification of SeP as candidate for the treatment of dry eye was shown in Text S1 and Figure S1. SeP was identified from fraction 32 fractionated by HiTrap Q HP column as most effective component for treatment of dry eye. Figure 2 showed the results after 3 weeks of SeP eye drops use. SeP eye drops were clearly effective for suppression of corneal irritation (Fig. 2a). The fluorescein score of corneas in normal rats (no removal of lacrimal gland) remained low, and PBS treatment did not suppress increased fluorescein scores in dry eye rat (Fig. 2b). The fluorescein scores of corneas treated with SeP eye drops were significantly lower than the scores following treatment with PBS, and the effect was SeP dose dependent (i.e., 50 µg/mL SeP eyedrops was more effective than 5 µg/mL) (Fig. 2c). Furthermore, SeP eye drops suppressed production of 8-OHdG in corneas (Fig. 2d). We think that the decreased fluorescein score seen with SeP eye drops was related to suppression of oxidative stress.

### Selenium deprivation *in vitro* induced oxidative stress

**Figure 3:**
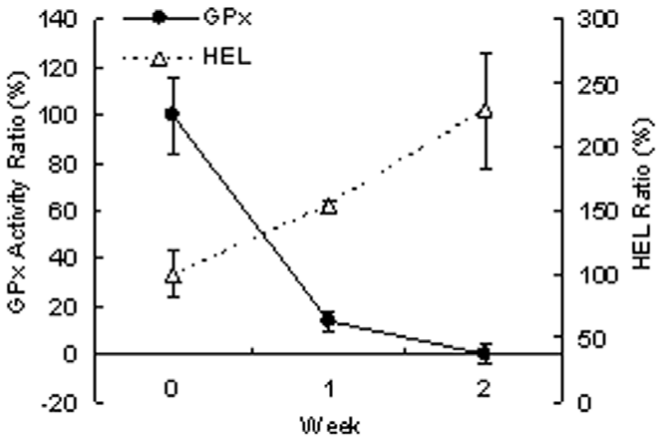
Effect of selenium deficiency on CEPI cells. Selenium deficiency-induced GPx activity and HEL production in CEPI cells. Open triangle shows the ratio of GPx activity and closed cycle shows ratio of HEL content. Data are mean ± S.D. (n = 6).

**Figure 4:**
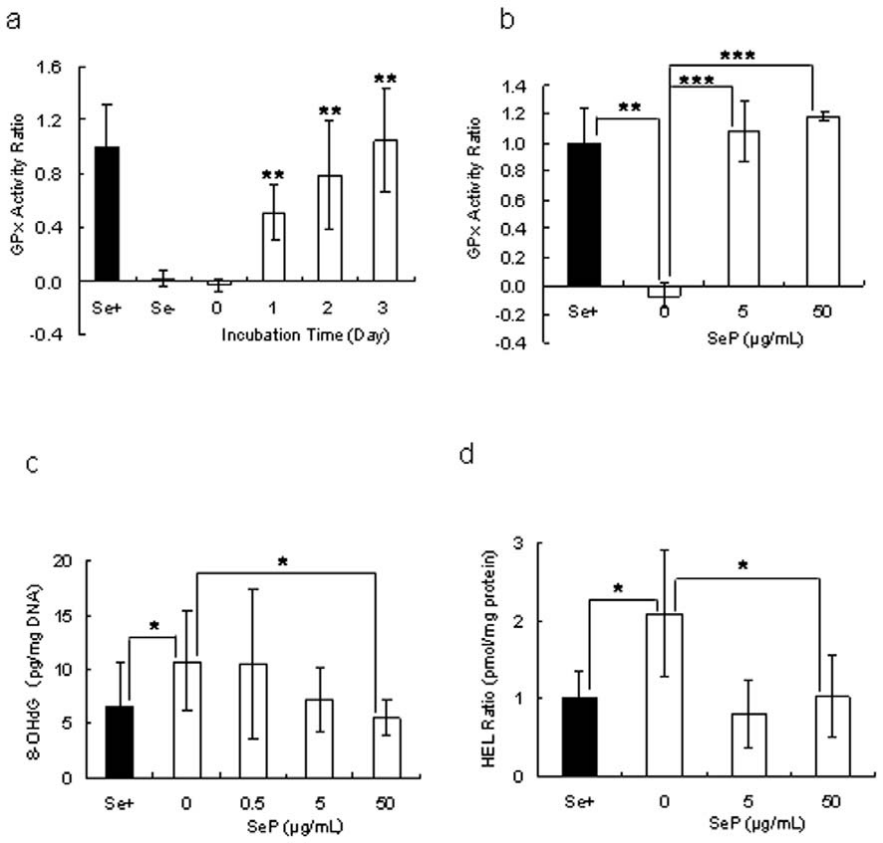
SeP addition suppresses oxidative stress in selenium-deficient CEPI cells. a: Recovery of GPx activity by addition of SeP. Fifty µg/mL SeP was added to culture medium and incubated for 0, 1, 2, or 3 days. ** indicates a significant difference from the result in Se-, *P*<0.01 (n = 8). b: Recovery of GPx activity by addition of SeP (5 or 50 µg /mL) and incubated for 3 days. **P<0.01 and ***P<0.005, respectively (n = 6). c, d: Suppression of production of 8-OHdG. (c, n = 8) and HEL (d, n = 5) in CEPI cells. SeP (0.5, 5 or 50 µg /mL) was added to medium and cells were incubated for 3 days. *P<0.05. All results are expressed as mean ± S.D. Dunnett's test was used to determine the significance of differences. Se+: GPx activity of CEPI cells cultivated in normal medium. Se-: GPx activity of CEPI cells cultivated in selenium-depleted medium.

**Figure 5:**
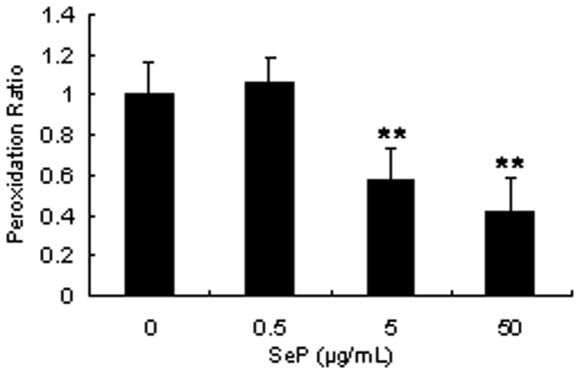
SeP suppresses lipid peroxidation in CEPI cells. The vertical axis shows peroxidation rate calculated from fluorescence intensity. Results are expressed as the mean ± S.D. (n = 4). Dunnett's test was used to determine the significance of differences. ** and *** indicate a significant difference from the result of 0 µg/mL SeP addition, *P*<0.01 and <0.005, respectively.

When CEPI cells were cultivated in selenium-deficient medium for 2 weeks, GPx activity was diminished (Fig. 3). In inverse proportion to GPx activity, the amount of HEL in CEPI cells increased, as GPx catalyzed hydrogen peroxide and lipid peroxide-producing HEL. GPx activity, which is a sign of selenium uptake, recovered following the addition of SeP to the culture medium. SeP was taken up by the CEPI cells; the selenium in SeP was then used to synthesize GPx (Fig. 4a, b). The concentration of SeP in Figure 4b was the same concentration as the eye drop experiment. We expected that SeP was already sufficient in the cells in 5 µ/ml SeP stimulation. Both 8-OHdG (Fig. 4c) and HEL (Fig. 4d) production were induced by selenium deprivation. The elevation of both of these oxidative stress markers was reduced by the addition of SeP. SeP uptake returned GPx activity to normal levels, and consequently 8-OHdG and HEL production were suppressed by GPx. Furthermore, SeP suppressed the lipid peroxidation in CEPI cells induced by cumene hydroperoxide (Fig. 5). Addition of 0.2 mM cumene hydroperoxide induced an increase in lipid peroxide from 34682±341 RFU (relative fluorescent unit) (mean ± S.D.) to 37027±1171 RFU (mean ± S.D.). Both 5 µM and 50 µM SeP concentrations significantly suppressed production of lipid peroxide.

### SeP decrease in tears from dry eye patients

**Figure 6:**
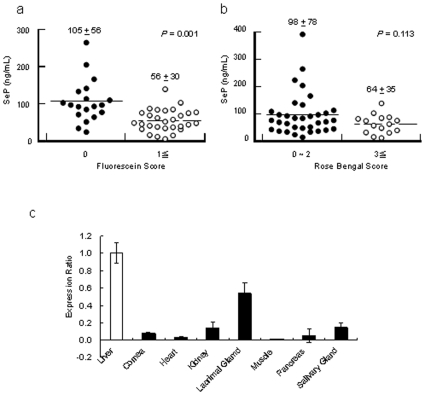
SeP in tears. a, b: Concentration of SeP in human tears. Fluorescein (a) and Rose Bengal (b) Scores are indices of dry eye diagnosis. Dry eye is indicated with fluorescein scores of ≥1, or when the Rose Bengal Score is ≥3. c: SeP expression in rat lacrimal glands. The vertical axis shows expression level of SeP in each tissue compared with liver. The expression level in the liver defined 1. All results are expressed as mean ± S.D.

Although SeP had been found in plasma, it was not known that SeP was present in human tears. We measured the concentration of SeP in human tears, finding SeP in tears of healthy volunteers at a concentration of 93.1±57.1 ng/mL (mean ± S.D.). We also measured the concentration of SeP in tears of dry eye patients, finding a mean concentration of 56.1±30.0 ng/mL (mean ± S.D.) (Fig. 6a). The concentration of SeP in non-dry eye patients was significantly higher than that in dry eye patients (*p* = 0.01), as evaluated by fluorescein score (Fig. 6a), and a similar result was obtained with Rose Bengal scoring (Fig. 6b).

### SeP expression in the rat lacrimal gland

To examine whether SeP was synthesized in the lacrimal gland or not, SeP mRNA expression was measured in lacrimal glands. RNA was extracted from rat lacrimal glands, liver, and other tissues because of the comparison of SeP expression levels in lacrimal gland and other tissues with that in liver, in which SeP in plasma is primarily produced. SeP expression in rat lacrimal glands was approximately 50% of in rat liver, and that in other tissues were below 15% (Fig. 6c). This result suggested SeP was extremely expressed in the lacrimal glands compared with in the other tissues.

## Discussion

One of the serum-derived candidates for the treatment of dry eye is serum albumin [18]. The concentration of albumin in serum is approximately 50 mg/mL and which was about 50% of the serum protein [1]. Serum albumin is a remarkably stable, and distributed widely in body fluids including tears [19]. The concentration of albumin in tears is same as serum and makes up approximately 1% of the protein in tears [1]. In our previous study, 50 mg/mL albumin was applied as eyedrops for patients with severe dry eye caused by Sjogren's syndrome [20]. The patients showed significant improvement in both their fluorescein and Rose Bengal scores, but the differences were not noted in tear break-up time or subjective symptoms.

Other possible candidates are tear components such as epidermal growth factor and retinoic acid in serum [1] as previously reported [21], which played an important role in the maintenance of the ocular surface. However these components may be useful for the treatment of dry eye, but they are not sufficiently efficacious; for this reason, we wanted to identify other serum-derived candidates.

The physiological role of selenium is probably its incorporation into selenocysteine (Sec), cysteine analogue with a sulfur atom replacing a selenium atom. The protein containing of Sec is called selenoprotein. Twenty-five selenoprotein genes are present in the human genome, but little beyond genomic data is known about many of them [22]. GPx was the first selenoprotein identified in mammals [23]. GPx consists of several isozymes and acts to protect cells from oxidative damage by catalyzing the reduction of H_2_O_2_, lipid hydroperoxides, and other organic peroxides. Since Sec residue is contained in the active site of GPx, Sec is thought to be essential for activity [24]. GPx is widely distributed in tissues of the body including the ocular surface [16], [25]. Figure 3 shows the reduction of GPx activity in CEPI cells cultivated in selenium-deficient medium. This reduction was caused by cellular Se exhaustion, because Sec residue is essential for GPx activity. Reduction of GPx activity reduce the capacity to prevent anti-oxidative damage and induced 8-OHdG and HEL accumulation in the cells.

SeP is selenium-transfer plasma glycoprotein [26] and is present in extracellular fluids such as plasma [27] and milk [28]. The principal source of SeP is thought to be the liver [29]. In this paper, we report that SeP found in tears (Fig. 6) and was synthesized in the lacrimal gland. SeP contains ten Sec residues; one Sec is located at the 40th residue in the N-terminal, and other residues are localized in the C-terminal. SeP is a bi-functional protein [30], as the Sec residue in the N-terminal shows GPx activity [31] and Sec residues in C-terminal are functioned as cellular selenium suppliers [32]. Although SeP did not reduce H_2_O_2_, it could reduce phospholipids hydroperoxide [33]. SeP blocked ultraviolet-induced cell death [33] (which is related to production of active oxygen) and fatty acid hydroperoxide-induced cell death [34].

Figure 2 shows improvement of dry eye by treatment with 50 µg/mL SeP. This effect is thought to be due to two functions of SeP, *i.e.* GPx activity and Sec supply activity. The function of SeP as selenium supplier may be more important than the function to reduce the effect of anti-oxidative stress on corneas. Attempts to measure the content of selenium in corneas of normal rats using X-ray absorption fine structure methods were unsuccessful because of the low content of selenium (data not shown). We hypothesized that reduction of tear volume secreted from the lacrimal gland reduced the selenium supply to the cornea from tears, and easily induced a shortage of selenium in the cornea. A shortage of selenium led to an increase in oxidative stress in corneal cells accompanied by diminished GPx activity (Fig. 3). It was shown in this (Fig. 1) and a previous paper [5] that oxidative stress increased in corneas of the dry eye rat model. SeP supply is thought to increase selenium content and, as a result, revive GPx activity in the cornea.

Selenium source of supply to cornea epithelium is probably SeP in the tears. Because SeP is expressed in the rat lacrimal gland (Fig. 6c), SeP in the tears is thought to synthesize and secreted from the lacrimal gland. It is supposed that highly expression level of SeP in the lacrimal gland is necessary for sufficient supply of selenium to the cornea. High concentrations of lipid peroxide were detected in dry eye patients compared with tears from normal controls [35]. Our results suggest that oxidative stress increases in the ocular surface of dry eye. In the tears from patients who have corneal damage, the SeP concentration was lower than control (*p* = 0.001, Fig. 6a). The content of selenium in the corneas may be reduced by the low level of SeP in the tears of those patients.

Ocular surface cells are damaged by physical and chemical factors which induce oxidative stress. SeP in tears physiologically protects the ocular surface against the influence of oxidative stressors.

## Supporting Information

Text S1 (0.03 MB DOC)

Figure S1 Identification of candidate for dry eye treatment from human plasma. a: Treatment effect of fraction 41-h on corneal epithelial erosion induced by ocular surface desiccation. Results are expressed as mean ± S.D (n = 6). Paired Student's t-test was used to determine the significance of differences. * indicates a significant difference from the result of PBS treatment, P<0.05. b: Fractionation of Fraction 41-h using HiTrap Q HP chromatography. The vertical axis shows ratio of DNA fragmentation of each fraction compared with HiTrap Q HP-applied solution (Fraction 41-h). c: SDS-PAGE pattern of Fraction 31. Marker: molecular weight marker; Fr32: Fraction 32.
(1.71 MB TIF)
