# Supplementary material for: Selenoprotein P Controls Oxidative Stress in Cornea
Source: PLoS One. 2010 Mar 29;5(3):e9911. doi: 10.1371/journal.pone.0009911 (PMC2847950; doi:10.1371/journal.pone.0009911)
Supplement: Text S1 — (0.03 MB DOC) [file pone.0009911.s001.doc]

**Supporting Information S1**

**Identification of candidates for the treatment of dry eye from serum**

Since autologous serum is useful for the treatment of severe dry eye, serum components may be potential candidates for the treatment of dry eye. We fractionated human plasma by column chromatographies to identify candidates for the treatment of dry eye. Conjunctival epithelial cell line, CCL-20.2 (CCL) was obtained from the American Type Culture Condition (Manassas, VA). When serum is removed from the culture medium, CCL undergoes apoptosis. This apoptosis can be protected by the addition of fractionated-plasma to medium. Therefore, we used ratio of apoptosis suppression in CCL caused by addition of fractionated-plasma to medium as the index of identification. Ratio of apoptosis was measured by using DNA fragmentation detection kit (Cell Death Detection ELISA kit, Roche Diagnostics GmbH, Mannheim, DE) referred to in a previous study [21].

Human plasma was fractionated by standard method**s**. The heparin column-absorbed fraction was used as a starting fraction, as it was most effective on suppression of DNA fragmentation in CCL. The heparin column-absorbed fraction was purified using 3 column chromatographies (anion exchange, cation exchange, and hydrophobic chromatography). Since Fraction 41-h fractionated by hydrophobic chromatography was most effective, we tried to estimate efficacy of this fraction on the treatment of dry eye using our dry eye rat model.

**Estimation of efficacy of partial purified serum using dry eye rat model**

The method was according to our previous study [36]. Male 3-week-old Sprague-Dawley rats (n = 6) were purchased from Tokyo Laboratory Animal Science Co., Ltd (Tokyo, Japan). After deep anesthesia, the eyelids of the rats were fixed (using adhesive agent) to keep their eyes open. The rats were placed in a desiccation room (as shown in the previous study [36]) for 8 hours. During desiccation, one eye of each rat was treated with 1 mg/mL Fraction 41-h, and the other eye was given a drop of PBS as the control. Ten ml of eye drops was administrated every hour for 8 hours. After eye drop administration, the ocular surfaces of all rats were photographed after applying a fluorescein solution under cobalt blue light to quantify the area of corneal epithelial erosion.

The erosion area treated with 1 mg/mL of Fraction 41-h was approximately half the size of the erosion seen with the PBS eye drop (Supplementary Fig. S1a). Since the treatment efficacy of Fraction 41-h was confirmed by this *in vivo* experiment, we attempted to search for a candidate for the dry eye treatment from Fraction 41-h using a mono Q column.

**Identification of candidate for the dry eye treatment derived from Fraction 41-h**

Fraction 41-h was further fractionated by HiTrap Q HP column (GE Healthcare Bio-Science Corp., Piscataway, NJ). Each fraction was estimated on suppression of DNA fragmentation of CCL and was applied on SDS-polyacrylamide gel electrophoresis (SDS-PAGE). Fractions 32 (fractionated by HiTrap Q column) effectively suppressed apoptosis of CCL induced by serum deprivation (Supplementary Fig. S1b). SDS-PAGE analysis of each fraction showed strongly stained-bands of high molecular weight (more than 50 kDa) in Fraction 34-39 (data not shown). In Fractions 32 and 33, these high molecular weight bands were weakly stained, and two of the weakly stained bands were detected near 32 kDa (Band a) and 24 kDa (Band b) (Supplementary Fig. S1c). Band b was removed from the gel, and identified as a fragment of selenoprotein P (SeP) by the Edman method.
