# Supplementary figures and images for: Selenoprotein P Controls Oxidative Stress in Cornea
Source: PLoS One. 2010 Mar 29;5(3):e9911. doi: 10.1371/journal.pone.0009911 (PMC2847950; doi:10.1371/journal.pone.0009911)

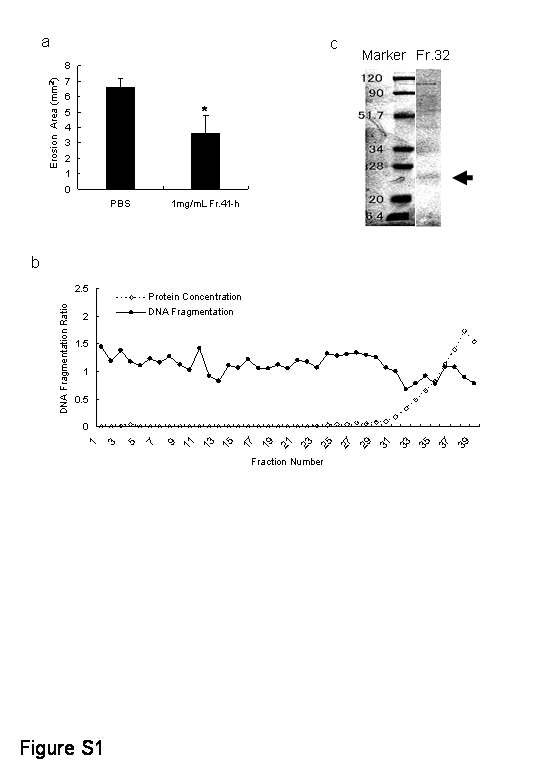

Supplement: Figure S1 — Identification of candidate for dry eye treatment from human plasma. a: Treatment effect of fraction 41-h on corneal epithelial erosion induced by ocular surface desiccation. Results are expressed as mean ± S.D (n = 6). Paired Student's t-test was used to determine the significance of differences. * indicates a significant difference from the result of PBS treatment, P<0.05. b: Fractionation of Fraction 41-h using HiTrap Q HP chromatography. The vertical axis shows ratio of DNA fragmentation of each fraction compared with HiTrap Q HP-applied solution (Fraction 41-h). c: SDS-PAGE pattern of Fraction 31. Marker: molecular weight marker; Fr32: Fraction 32. (1.71 MB TIF) [file pone.0009911.s002.tif]
